# Application of the Specified Stress Method to Crack Propagation Analysis in Reinforced Concrete Members

**DOI:** 10.3390/ma19153231

**Published:** 2026-07-29

**Authors:** Xiaoqing Zhang, Jialin Wang, Zhijian Yi, Tuo Zhang

**Affiliations:** School of Civil Engineering, Chongqing Jiaotong University, Chongqing 400074, China; kindzhxq@163.com (X.Z.);

**Keywords:** specified stress method, reinforced concrete member cracking, numerical simulation

## Abstract

Reinforced concrete (RC) structures are susceptible to crack initiation and propagation during service, making accurate numerical simulation of crack behavior essential for assessing structural durability and safety. Current numerical approaches for simulating concrete cracking include smeared/continuum approaches, extended finite element method (XFEM), phase-field methods, and meso-mechanical models. In particular, smeared/continuum approaches (e.g., smeared crack and plastic-damage models such as CDP) indirectly reflect cracking through diffusive damage fields without providing explicit geometric information on crack locations and propagation paths. The XFEM module in commercial software is further restricted to first-order elements and encounters difficulties in simulating multi-crack propagation. These limitations indicate that further development of complementary crack-simulation frameworks is warranted. To this end, this paper presents a cracking simulation framework for RC members within the theoretical framework of the Specified Stress Method, adopting an adaptive degree-of-freedom strategy to balance computational accuracy and efficiency. The method introduces inelastic strain as an additional unknown and establishes a variational principle and the corresponding virtual work equation. Concrete cracking is described by specifying the stress on the crack plane to zero, so that the crack-surface stress remains zero after cracking, thereby avoiding the issue of damage reversibility and improving computational convergence. The method requires neither a predefined crack path nor remeshing after cracking. Unlike smeared/continuum approaches that rely on diffusive damage fields, the crack propagation paths, distribution characteristics, and evolution of multiple cracks are characterized through the spatial distribution of cracked integration points within the finite element mesh. In the present implementation, crack initiation is governed by the maximum tensile stress criterion, and a linear elastic constitutive model is adopted for concrete as a deliberate simplification to establish and verify the core computational mechanism of the framework. The proposed method was examined through three numerical examples. First, comparison with theoretical solutions confirmed the algorithm’s correctness in simulating cracking in heterogeneous RC tension members. Second, comparison with experimental results demonstrated qualitatively consistent crack propagation trends and load–displacement responses for RC beams under mixed-mode cracking; the calculated ultimate load of the plain concrete beam is lower than the experimental value, which is attributable to the use of the maximum tensile stress criterion without fracture energy considerations, and certain crack morphology deviations are observed due to the neglect of reinforcement–concrete bond-slip. Third, a multi-crack simulation of an under-reinforced RC beam showed that, whereas the XFEM module in ABAQUS captures only a single dominant crack near the mid-span, the proposed algorithm predicts multiple distributed cracking zones on both sides of the mid-span, qualitatively consistent with the typical flexural cracking behavior of under-reinforced RC beams; the algorithm also supports second-order elements (e.g., C3D20R) unavailable in the ABAQUS XFEM implementation. While the method is still in an exploratory stage, these results confirm the feasibility and potential of the Specified Stress Method as a complementary framework for RC cracking simulation, providing a basis for further development.

## 1. Introduction

Reinforced concrete (RC) structures are widely utilized across various engineering disciplines, including civil engineering (for bridges, buildings, tunnels, and hydraulic ports), nuclear energy engineering (for reactor pressure vessel enclosures), marine engineering (for large-scale offshore platforms and oil tankers), and mechanical manufacturing (for ultra-heavy-tonnage hydraulic press frames). The widespread use of RC structures is primarily due to their exceptional mechanical properties and superior technical-economic performance. A defining characteristic of RC structures is their operation with cracks, which significantly affects their durability and in-service performance. The initiation and propagation of these cracks are critical to maintaining structural integrity. Numerical simulation offers several advantages, including non-destructive analysis, cost-effectiveness, repeatability, and the ability to circumvent limitations associated with experimental measurements. The advancement of cracking models has provided effective tools for investigating crack behavior in RC structures. These models facilitate comprehensive simulations of the entire lifecycle of crack initiation, propagation, and structural failure, thereby offering significant benefits over traditional methods.

In 1967, Ngo and Scordelis [1] introduced the groundbreaking discrete crack model for RC structures. Since then, researchers have developed numerous effective cracking models, including the smeared crack model [2], fictitious crack model [3], band crack model [4], and fracture models based on the extended finite element method (XFEM) [5,6], along with their improved and hybrid derivatives [7,8]. Among these approaches, the smeared crack model has been widely adopted because it is naturally compatible with continuum mechanics [9,10]. By “smearing” the effects of cracking at the element level, this model captures cracking behavior indirectly through material constitutive relations. It requires neither a predefined crack path nor mesh reconstruction, thereby providing a favorable balance between computational efficiency and accuracy. In engineering practice, the Concrete Damaged Plasticity (CDP) model implemented in commercial software such as ABAQUS represents one of the most widely adopted approaches for nonlinear analysis of RC structures. However, the CDP model characterizes concrete cracking indirectly through contour plots of maximum principal plastic tensile strain, without providing explicit geometric information on individual crack locations, orientations, or widths—information that is critical for structural durability assessment, crack width control, and post-cracking behavior evaluation.

In contrast, XFEM commonly uses enrichment functions together with crack representation techniques (e.g., level-set-based descriptions) to explicitly define crack locations and propagation paths, determining crack growth directions based on principles of fracture mechanics. Since its introduction, XFEM has been extensively applied to crack analysis in concrete structures [11,12], with commercial software platforms like ABAQUS and LS-DYNA already incorporating XFEM-based fracture analysis modules.

Since the 1980s, nonlocal regularization methods have been introduced into numerical simulations of concrete cracking to overcome the mesh dependence inherent in traditional local damage theories in the strain-softening regime. A commonly used regularization strategy incorporates nonlocal effects into the constitutive equations via a characteristic length parameter that represents the interaction range of the material microstructure. This treatment provides stable and mesh-independent numerical solutions upon refinement within the softening regime. Representative approaches include integral nonlocal models [13] and gradient-enhanced damage models [14]. In addition, phase-field approaches to fracture have gained considerable momentum in recent years [15,16]. Based on variational principles, these methods incorporate fracture energy into the total potential energy functional and can naturally represent complex fracture patterns—such as crack initiation, branching, and intersection—without requiring a predefined crack path or specialized crack-tracking procedures.

With advances in computational capabilities and the ongoing development of meso-scale mechanics theories, meso-scale models have provided a new paradigm for elucidating the intrinsic relationship between concrete meso-structure and macroscopic behavior. The Lattice Discrete Particle Model (LDPM), a representative meso-scale approach, explicitly describes the interactions among coarse aggregate particles and has shown excellent performance in predicting the failure of cementitious materials [17,18,19]. It has also been extended to complex loading conditions and a variety of material forms [20,21].

However, when LDPM is directly applied to full-scale structural analyses, its refined representation results in substantially increased computational cost and memory requirements [22]. To address this limitation, Rezakhani et al. [23,24] developed a multiscale analysis framework for plain and reinforced concrete based on the asymptotic expansion homogenization (AEH) method. Within this framework, Zhu et al. [25] simulated the fracture evolution process in concrete foundations and developed a multiscale LDPM that integrates multiple long short-term memory (LSTM) networks within a digital twin framework for structural damage identification and condition assessment [26].

Despite the distinct features of the aforementioned methods, they still exhibit certain limitations. Traditional smeared crack models, nonlocal regularization approaches, and phase-field methods do not provide explicit sharp crack geometry (i.e., a discrete discontinuity surface) and mainly rely on diffusive damage/phase fields. In addition, these approaches typically involve strongly nonlinear solution procedures and may suffer from convergence difficulties and high computational costs in post-cracking regimes [27]. Although XFEM can represent discontinuous displacement fields, it remains challenging to accurately model complex fracture patterns such as multi-crack propagation and crack intersection. Moreover, its implementation in existing commercial software is largely restricted to linear elastic fracture mechanics frameworks and low-order elements.

In conventional gradient-enhanced damage models, damage irreversibility is typically enforced through history variables; however, the non-local nature of the equivalent strain field may still lead to spurious damage spreading into unloading regions, resulting in physically unrealistic damage evolution and mechanical responses [28]. Phase-field methods typically entail high computational costs and often require extremely fine mesh refinement in the crack region to meet accuracy requirements [29].

Meso-scale models involve an inherent trade-off between modeling accuracy and computational efficiency: accurately resolving the interfacial transition zone (ITZ) requires a very high mesh density, whereas simplifying assumptions may introduce deviations in damage prediction [30,31].

In summary, existing methods still have limitations with respect to simulation accuracy, computational efficiency, and geometric crack characterization, indicating that further exploration of crack-simulation approaches is warranted. In particular, a unified numerical framework that is simultaneously designed to provide direct geometric characterization of crack distribution, capable of representing multi-crack propagation, structured to inherently avoid damage reversibility, and compatible with higher-order finite elements has yet to be fully established for RC cracking simulation.

The Specified Stress Method, originally developed in [32], offers a complementary computational framework with clear mechanical interpretation for simulating the cracking behavior of RC members. It should be emphasized that the novelty of the present work lies primarily in the computational formulation and its finite element implementation, rather than in a fundamentally new constitutive description of concrete fracture: the constitutive behavior of concrete is simplified to linear elasticity, crack initiation is governed by the classical maximum tensile stress criterion, and what distinguishes the proposed approach is the manner in which the cracking condition is enforced numerically within a variational framework. The method is formulated to address problems in finite element analysis where stress prescription conditions are imposed—for instance, the case in which the normal stress on a crack surface is released to zero upon cracking of concrete. It introduces inelastic strain as an additional unknown and establishes a variational principle and virtual work equation in which both displacement and the unknown inelastic strain serve as independent variables. When the Specified Stress Method is applied to simulate concrete cracking, the principal stress along the crack plane can be directly set to zero upon crack formation, while the material in the direction perpendicular to the crack plane remains capable of carrying load. For an existing crack surface and in the absence of newly formed crack planes, the crack-plane stress constraint can be enforced in a straightforward manner, without the need for repeated iterative corrections on that crack plane. For crack propagation problems, the method requires neither a predefined crack path nor remeshing after cracking; instead, the crack propagation trajectory, spatial distribution, and evolution of multiple cracks can be characterized directly through the distribution of cracked integration points within the finite element mesh. Since the stress on a crack surface is released to zero and maintained at zero throughout the analysis, the method eliminates the issue of damage reversibility, thereby ensuring favorable numerical stability and convergence in the cracking simulation. At the same time, the structural stiffness matrix retains its symmetric and sparse character, which facilitates finite element implementation and computational efficiency.

To clearly position the scientific contribution of this study, it should be noted that the present work does not propose a new fracture mechanics theory or a new constitutive model for concrete. Rather, the contribution of this paper is threefold: (i) the application of the previously developed Specified Stress Method [32]—originally formulated as a general finite element approach for problems involving stress specified conditions—to the specific context of concrete cracking; (ii) the implementation of this method into a complete finite element framework for reinforced concrete crack simulation, including the derivation of the corresponding formulation, the treatment of crack-plane stress release, and the development of an adaptive degree-of-freedom strategy, realized in a self-developed C++ framework using Microsoft Visual Studio 2022; and (iii) an initial validation of the computational framework through comparison with theoretical solutions, experimental results, and the XFEM module in ABAQUS. In this sense, the present study serves as a feasibility demonstration of the Specified Stress Method for RC cracking analysis, providing a computational foundation upon which more advanced constitutive models and fracture mechanics-based criteria can subsequently be incorporated.

In light of the above, this study develops a finite element analysis method for simulating the cracking behavior of RC members within the specified stress theoretical framework, incorporating an adaptive degree-of-freedom strategy to balance computational accuracy and efficiency. On the basis of a detailed derivation of the finite element formulation, a corresponding numerical program is implemented in C++ to simulate crack initiation, crack propagation paths, and the evolution of multiple cracks in RC members. In the current implementation, crack initiation is governed by the maximum tensile stress criterion: cracking is assumed to occur when the maximum principal tensile stress at an integration point reaches the tensile strength of the material. This criterion does not account for fracture energy dissipation during crack propagation. Incorporation of fracture mechanics-based criteria considering fracture energy is identified as an important direction for future refinement of the framework.

To verify and examine the effectiveness and applicability of the proposed method, three numerical examples are considered: the first two are compared against theoretical solutions and experimental results, respectively, while the third assesses the multi-crack simulation capability of the proposed algorithm by comparing crack distribution results with those from the XFEM module in ABAQUS under identical modeling conditions—without reference to experimental data—and shows that, whereas ABAQUS XFEM captures only a single dominant crack near the mid-span, the proposed algorithm predicts multiple distributed cracking zones on both sides of the mid-span, a result qualitatively consistent with the well-known flexural cracking behavior of under-reinforced RC beams; the algorithm also supports second-order elements (e.g., C3D20R) that are unavailable in the ABAQUS XFEM implementation. The examples cover representative problems including cracking in heterogeneous concrete materials, mixed-mode cracking behavior in RC beams, and crack development and multiple-crack evolution in under-reinforced beams. The results indicate that the proposed method is capable of capturing the fundamental cracking features of RC members—including crack initiation, crack propagation trends, and multi-crack distributed evolution—while providing qualitatively reasonable load–displacement responses. This work demonstrates the feasibility and potential of the Specified Stress Method as a complementary framework for RC cracking simulation, with scope for further refinement through the incorporation of nonlinear constitutive models, fracture mechanics-based cracking criteria, and reinforcement–concrete bond-slip effects.

## 2. Theoretical Basis of the Specified Stress Method

**Figure 1:**
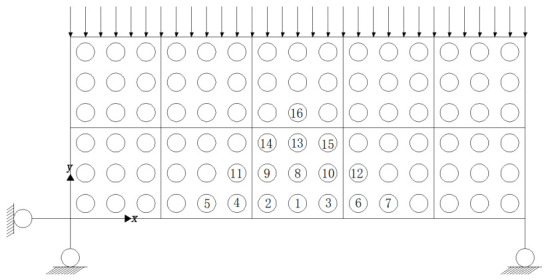
Simulation of the cracking process using the specified stress method.

Based on the theoretical framework of the Specified Stress Method developed in [32], the procedure for simulating cracking is outlined in detail as follows. A two-dimensional model (as shown in Figure 1) is considered, where each mesh element is analyzed using nine integration points. Structural stress analysis indicates that cracking typically initiates as longitudinal cracks in the *y*-direction. In this context, the integration point subjected to the maximum normal stress in the *x*-direction—corresponding to the maximum principal stress—becomes the most vulnerable to crack initiation. Initially, the normal stress at this critical integration point is specified as zero. Subsequently, the stresses at the adjacent integration points are calculated. The computed results can lead to three distinct scenarios:

In the first scenario, if none of the five adjacent integration points experiences stress that exceeds the cracking threshold, the model is considered free of cracks, and the computation process concludes.

In the second scenario, if only one of the adjacent integration points surpasses the cracking stress, this point is identified as the location of the crack. The stress in the direction of the maximum principal stress at this cracked point is then set to zero. An iterative recalculation of stresses for all integration points in the model is performed. This iterative process continues until no integration point exhibits stress levels exceeding the cracking threshold, at which point the computation concludes.

In the third scenario, if two to five of the adjacent integration points exceed the cracking stress, the integration point with the highest stress is designated as the cracked location. Similar to the previous scenario, the stress in the direction of the maximum principal stress at this point is prescribed as zero, followed by an iterative recalculation of stresses for all integration points. This iterative procedure persists until all integration points in the model demonstrate stress levels below the cracking threshold, concluding the computation.

This systematic approach allows for a comprehensive analysis of cracking behavior in the model, effectively capturing the progression of cracks through the specified stress method.

It is worth emphasizing that the formulation presented in this section represents the current stage of an ongoing, long-term development of the Specified Stress Method originally established in [32]. The prior work provides the general theoretical basis for finite element problems involving stress specified, whereas the present manuscript focuses on its computational specialization and implementation for reinforced concrete crack simulation. In particular, the contribution here is the derivation of an extended displacement-strain relationship and the corresponding extended stiffness matrix and load vectors that enable enforcing crack-plane stress release within a standard finite element framework. Therefore, the developments reported herein should be viewed as a continuation and application-oriented extension of the Specified Stress Method rather than the introduction of a fundamentally new crack model.

### 2.1. Displacement Mode

The element displacement mode used in conventional finite element methods follows the basic form below:

(1) $$u(x)\;=\;\overset{n}{\underset{i=1}{\Sigma }}N_{i}u_{i}\;=\;Nu_{e}$$

where u and x denote the displacement vector and coordinate vector of the element, respectively; $N_{i}$ represents the interpolation shape function matrix of node i; $u_{i}$ is the DOF vector of node i; N denotes the global interpolation shape function matrix of the element; $u_{e}$ is the nodal DOF vector of the element.

In accordance with the definition of the specified stress method [32], the total strain ε at any point within an element can be decomposed into three components: elastic strain $\varepsilon_{e}$, known inelastic strain $\varepsilon_{p}$ (induced by factors such as temperature variation or plastic deformation), and unknown inelastic strain $\varepsilon_{0}$. This decomposition relationship is mathematically expressed as

(2) $$\varepsilon =\varepsilon_{e}+\varepsilon_{0}+\varepsilon_{p}$$

It is assumed that the unknown inelastic strain $\varepsilon_{0}$ within the element can be parameterized by a set of unknown independent parameters $u_{0}$, as follows:

(3) $$\varepsilon_{0}\;={\;B}_{0}u_{0}$$

where $B_{0}$ denotes the generalized geometric matrix that correlates the unknown inelastic strain with the independent parameters within the element.

By substituting the geometric equation $\varepsilon \;=\;Bu_{e}$ (where B is the conventional geometric matrix) and Equation (3) into Equation (2), the elastic strain at any point within the element is derived as

(4) $$\varepsilon_{e}=\varepsilon -\varepsilon_{0}-\varepsilon_{p}\;\;=\left[B-{\;B}_{0}\right]\left\{\begin{array}{c} u_{e}\\ u_{0} \end{array}\right\}-\varepsilon_{p}\;=\overset{–}{\;B}\overset{–}{u}-\varepsilon_{p}$$

where

(5) $$\overset{–}{B}\;=\left[B-B_{0}\right]$$

is the extended geometric matrix; and

(6) $$\overset{–}{u}\;=\left\{\begin{array}{c} u_{e}\\ u_{0} \end{array}\right\}$$

represents the extended “displacement” vector based on the specified stress method.

### 2.2. Governing Equation

To establish the governing equation for the specified stress method, we start with the virtual strain energy formulation for specified stress problems, as referenced in [26]:

(7) $$\delta U\;=\;\int_{V}\sigma \delta \varepsilon_{e}\;dV\;+\;\int_{V_{\sigma }}\sigma_{0}\delta \varepsilon_{0}dV$$

where V represents the solution domain of the problem; σ denotes the elastic stress; $\delta \varepsilon_{e}$ is the virtual elastic strain; $V_{\sigma }$ refers to the specified stress region; $\sigma_{0}$ indicates the inelastic stress; and $\delta \varepsilon_{0}$ corresponds to the virtual inelastic strain.

Consider the variation in the previously derived equations for strain decomposition [Equation (4)] and the parameterization of the unknown inelastic strain [Equation (3)]. Substitute these varied expressions into the virtual strain energy formula [Equation (7)]. This substitution yields the updated virtual strain energy expression tailored to the specified stress method:

(8) $$\delta U\;=\;\int_{V}\delta {\overset{–}{u}}^{T}{\overset{–}{B}}^{T}\sigma dV\;+\;\int_{V_{\sigma }}\delta u_{0}^{T}B_{0}^{T}\sigma_{0}dV$$

Consistent with the classical finite element method, the virtual work done by the equivalent nodal loads of the element (i.e., the work done by external loads on the model) remains expressed as

(9) $$\delta W=\delta u_{e}^{T}F_{e}$$

where $\delta u_{e}$ is the virtual element nodal displacement and $F_{e}$ is the equivalent load.

By substituting Equations (8) and (9) into the virtual work equation (δU = δW), while considering the arbitrariness of $\delta \overset{–}{u}$ and introducing the physical relationship $\sigma =\;D\varepsilon_{e}$, the discretized virtual work equation is obtained as follows:

(10) $$\int_{V}{\overset{–}{B}}^{T}D\overset{–}{B}\overset{–}{u}dV\;=\;\left\{\begin{array}{c} F_{e}\\ -\int_{V_{\sigma }}B_{0}^{T}\sigma_{0}dV \end{array}\right\}\;+\;\int_{V}{\overset{–}{B}}^{T}D\varepsilon_{p}dV$$

Expressing this equilibrium equation in a concise matrix form:

(11) $${\overset{–}{K}}_{e}\overset{–}{u}\;=\;{\overset{–}{F}}_{e}$$

where ${\overset{–}{K}}_{e}$ is the extended stiffness matrix of the element based on the specified stress method, defined as

(12) $${\overset{–}{K}}_{e\;}=\int_{V}{\overset{–}{B}}^{T}D\overset{–}{B}dV$$

This matrix integrates the contributions of both conventional nodal displacements and unknown inelastic strain parameters, reflecting the overall stiffness of the element under specified stress conditions.

And ${\overset{–}{F}}_{e}$ is the extended equivalent nodal load vector based on the specified stress method, defined as

(13) $${\overset{–}{F}}_{e}=\left\{\begin{array}{c} F_{e}\\ -\int_{V_{\sigma }}B_{0}^{T}\sigma_{0}dV \end{array}\right\}+\int_{V}{\overset{–}{B}}^{T}D\varepsilon_{p}dV$$

Finally, substituting the expression for the extended geometric matrix [Equation (5)] into the extended stiffness matrix formula [Equation (12)], we obtain a more detailed form for computation:

(14) $${\overset{–}{K}}_{e}\;=\;\int_{V}{\left[B\;\;-B_{0}\right]}^{T}D\left[B-B_{0}\right]dV\;=\;\int_{V}\left[\begin{array}{cc} B^{T}\mathrm{DB} & {-B}^{T}DB_{0}\\ {-B}_{0}^{T}\mathrm{DB} & B_{0}^{T}DB_{0} \end{array}\right]dV$$

## 3. Modeling of Steel-Concrete Bond

The reinforcement and concrete are connected at the degree-of-freedom level. To strike a balance between computational efficiency and modeling accuracy, the algorithm proposed in this study assumes a perfect bond between the reinforcement and concrete, meaning that no relative slip occurs at the steel-concrete interface. This assumption inevitably limits the capability of the present framework to reproduce bond–slip induced cracking phenomena and crack patterns strongly governed by reinforcement slip. The limitation is discussed later in Example 2 and will be considered in future developments. The reinforcement is modeled using spatial truss elements, which effectively capture axial tension and compression forces without introducing unnecessary bending stiffness. In contrast, concrete is represented as three-dimensional solid elements to accurately depict its nonlinear behavior, including phenomena such as cracking, crushing, and volumetric deformation. For clarity in formulation, the nodes of the reinforcement truss elements are designated as “internal nodes”, which are located within the domain of the concrete elements. The concrete elements that encompass these internal nodes are referred to as parent elements.

In the global spatial coordinate system, the displacement of any internal node *k* of the reinforcement truss element can be interpolated from the nodal displacements of its parent concrete element. This interpolation leverages the shape function of the parent concrete element to ensure compatibility between the reinforcement and concrete displacement fields, and it is mathematically expressed as

(15) $$u^{k}=\left\{\begin{array}{c} u_{1}^{k}\\ u_{2}^{k}\\ u_{3}^{k} \end{array}\right\}=Nu_{i}$$

Equation (15) can be expressed in the following block matrix form:

(16) $$u^{k}\;=\;\left[\begin{array}{ccc} S_{1}^{m_{1}} & 0 & 0\\ 0 & S_{2}^{m_{2}} & 0\\ 0 & 0 & S_{3}^{m_{3}} \end{array}\right]\left\{\begin{array}{c} u_{e}^{m_{1}}\\ u_{e}^{m_{2}}\\ u_{e}^{m_{3}} \end{array}\right\}$$

where $S_{i}^{m_{i}}(i\;=\;1,2,3)$ denotes the *i*-th row of the shape function matrix N of the parent element $m_{i}$, and $u_{e}^{m_{i}}\;(i\;=\;1,2,3)$ is the DOF vector of the parent element $m_{i}$. It is important to note that an internal node of the reinforcement can only reside within one parent concrete element; thus, the parent element DOF vectors $u_{e}^{m_{i}\;}(i\;=\;1,2,3)$ corresponding to each component of the same internal node’s displacement are identical (i.e., the parent element index *m_i_* is consistent for all *i*, and the DOF vectors are repeated for notational completeness).

For a typical reinforcement truss element with two nodes *k* and *l*, the relationship between the global DOF vector $u^{S}$ of the entire reinforcement element and the nodal DOF vectors of the parent concrete elements can be extended from Equation (15) and expressed in a concise block matrix form:

(17) $$u^{S}\;=\;\left\{\begin{array}{c} u^{k}\\ u^{l} \end{array}\right\}\;=\;\left[\begin{array}{c} \begin{array}{cc} \begin{array}{cc} S_{1}^{m_{1}} & 0 \end{array} & \begin{array}{cc} \cdots & 0 \end{array} \end{array}\\ \begin{array}{cc} \begin{array}{cc} 0 & S_{2}^{m_{2}} \end{array} & \begin{array}{cc} \cdots & 0 \end{array} \end{array}\\ \begin{array}{cc} \begin{array}{ll} \text{⋮}\;\; & \text{⋮} \end{array} & \begin{array}{cc} \;\;\;\ddots & \;\;\;\vdots \end{array} \end{array}\\ \begin{array}{cc} \begin{array}{cc} 0 & \;0 \end{array} & \begin{array}{cc} \cdots & S_{6}^{m_{6}} \end{array} \end{array} \end{array}\right]\left\{\begin{array}{c} u_{e}^{m_{1}}\\ u_{e}^{m_{2}}\\ \vdots \\ u_{e}^{m_{6}} \end{array}\right\}$$

where ${\left\{u_{e}^{m_{1}}\;u_{e}^{m_{2}}\;\cdots u_{e}^{m_{6}}\right\}}^{T}$ is the generalized DOF vector of the reinforcement element.

In the local nodal coordinate system of the reinforcement element, the mechanical equilibrium between the nodal DOF vector $u^{S}$ and the equivalent nodal force vector $F^{S}$ follows the standard truss element stiffness relation:

(18) $$K^{S}u^{S}\;=\;F^{S}$$

Substituting the transformation relation [Equation (17)] into this equilibrium equation and applying the principle of virtual work to ensure consistency with the parent concrete elements, the equilibrium equation of the reinforcement element is rederived in terms of the generalized DOF vector of the concrete elements. After simplification, this extended equilibrium equation takes the form:

(19) $${\overset{–}{K}}^{S}\left\{\begin{array}{c} u_{m_{1}}^{e}\\ u_{m_{2}}^{e}\\ \vdots \\ u_{m_{6}}^{e} \end{array}\right\}\;=\;{\overset{–}{F}}^{S}$$

where

(20) $${\overset{–}{K}}^{S}={\left[\begin{array}{c} \begin{array}{cc} \begin{array}{cc} S_{1}^{m_{1}} & 0 \end{array} & \begin{array}{cc} \cdots & 0 \end{array} \end{array}\\ \begin{array}{cc} \begin{array}{cc} 0 & S_{2}^{m_{2}} \end{array} & \begin{array}{cc} \cdots & 0 \end{array} \end{array}\\ \begin{array}{cc} \begin{array}{ll} \vdots \;\; & \vdots \end{array} & \begin{array}{cc} \ddots & \;\;\vdots \end{array} \end{array}\\ \begin{array}{cc} \begin{array}{cc} 0 & \;0 \end{array} & \begin{array}{cc} \cdots & S_{6}^{m_{6}} \end{array} \end{array} \end{array}\right]}^{T}K\left[\begin{array}{c} \begin{array}{cc} \begin{array}{cc} S_{1}^{m_{1}} & 0 \end{array} & \begin{array}{cc} \cdots & 0 \end{array} \end{array}\\ \begin{array}{cc} \begin{array}{cc} 0 & S_{2}^{m_{2}} \end{array} & \begin{array}{cc} \cdots & 0 \end{array} \end{array}\\ \begin{array}{cc} \begin{array}{cc} \vdots \;\; & \vdots \end{array} & \begin{array}{cc} \ddots & \;\;\vdots \end{array} \end{array}\\ \begin{array}{cc} \begin{array}{cc} 0 & \;0 \end{array} & \begin{array}{cc} \cdots & S_{6}^{m_{6}} \end{array} \end{array} \end{array}\right]\;=\left[\begin{array}{c} \begin{array}{cc} \begin{array}{cc} {{\left(S_{1}^{m_{1}}\right)}^{T}K_{1,1}S}_{1}^{m_{1}} & {{\left(S_{1}^{m_{1}}\right)}^{T}K_{1,2}S}_{2}^{m_{2}} \end{array} & \begin{array}{cc} \cdots & {{\left(S_{1}^{m_{1}}\right)}^{T}K_{1,6}S}_{6}^{m_{6}} \end{array} \end{array}\\ \begin{array}{cc} \begin{array}{cc} {{\left(S_{2}^{m_{2}}\right)}^{T}K_{2,1}S}_{1}^{m_{1}} & {{\left(S_{2}^{m_{2}}\right)}^{T}K_{2,2}S}_{2}^{m_{2}} \end{array} & \begin{array}{cc} \cdots & {{\left(S_{2}^{m_{2}}\right)}^{T}K_{2,6}S}_{6}^{m_{6}} \end{array} \end{array}\\ \begin{array}{cc} \begin{array}{cc} \vdots \;\; & \vdots \;\; \end{array} & \begin{array}{rr} \;\;\ddots & \;\;\vdots \end{array} \end{array}\\ \begin{array}{cc} \begin{array}{cc} {{\left(S_{6}^{m_{6}}\right)}^{T}K_{6,1}S}_{1}^{m_{1}} & \;{{\left(S_{6}^{m_{6}}\right)}^{T}K_{6,2}S}_{2}^{m_{2}} \end{array} & \begin{array}{cc} \cdots & {{\left(S_{6}^{m_{6}}\right)}^{T}K_{6,6}S}_{6}^{m_{6}} \end{array} \end{array} \end{array}\right]$$

(21) $${\overset{–}{F}}^{S}={\left[\begin{array}{c} \begin{array}{cc} \begin{array}{cc} S_{1}^{m_{1}} & 0 \end{array} & \begin{array}{cc} \cdots & 0 \end{array} \end{array}\\ \begin{array}{cc} \begin{array}{cc} 0 & S_{2}^{m_{2}} \end{array} & \begin{array}{cc} \cdots & 0 \end{array} \end{array}\\ \begin{array}{cc} \begin{array}{cc} \vdots \;\; & \vdots \end{array} & \begin{array}{cc} \;\;\ddots & \;\;\vdots \end{array} \end{array}\\ \begin{array}{cc} \begin{array}{cc} 0 & \;0 \end{array} & \begin{array}{cc} \cdots & S_{6}^{m_{6}} \end{array} \end{array} \end{array}\right]}^{T}F^{S}=\left\{\begin{array}{c} {\left(S_{1}^{m_{1}}\right)}^{T}F_{1}^{S}\\ {\left(S_{2}^{m_{2}}\right)}^{T}F_{2}^{S}\\ \vdots \\ {\left(S_{6}^{m_{6}}\right)}^{T}F_{6}^{S} \end{array}\right\}$$

The assembly of the extended stiffness matrix ${\overset{–}{K}}^{S}$ into the global finite element stiffness matrix follows a straightforward rule: for each submatrix ${{\left(S_{i}^{m_{i}}\right)}^{T}K_{i,j}S}_{j}^{m_{j}}$, the row positions in the global matrix are determined by the DOF indices of the parent concrete element $u_{m_{i}}^{e}$, and the column positions are determined by the DOF indices of $u_{m_{j}}^{e}$. Similarly, each term ${\left(S_{i}^{m_{i}}\right)}^{T}F_{i}^{S}$ from the extended equivalent nodal force vector ${\overset{–}{F}}^{S}$ is added to the corresponding entries in the global load vector, aligned with the DOF indices of $u_{m_{i}}^{e}$. This assembly process seamlessly integrates the reinforcement’s mechanical contribution into the global model, ensuring consistent equilibrium between steel and concrete under external loads.

## 4. Structural Computation Reduction

Based on the preceding theoretical analysis, the solution procedure for cracking problems in RC members using the proposed approach is as follows: first, appropriate independent parameters $u^{0}$ are selected according to the structural mechanical behavior and computational requirements (in this study, $u^{0}$ is defined as the inelastic strain at element integration points for subsequent numerical examples) to form the extended displacement vector of each element. Next, the element equilibrium equation [Equation (11)] is assembled into the global equilibrium equation of the structure. Finally, the extended stiffness matrix ${\overset{–}{K}}^{S}$ of the reinforcement is integrated into the corresponding DOF positions in the global matrix, and the combined system of equations is solved to obtain the structural response.

However, practical computational experience reveals a significant challenge: the number of DOFs in the extended element stiffness matrix is nearly double that of the original matrix, even when reduced integration is employed. This leads to a substantial increase in the total number of DOFs during global structural analysis, with the increment becoming more pronounced as the element mesh is refined—directly compromising computational efficiency. For illustrative purposes, consider the use of spatial 20-node solid elements: the original element stiffness matrix has 20 × 3 = 60 DOFs (3 displacement components per node). When employing a 2 × 2 × 2 Gaussian integration scheme, which results in eight integration points per element, the extended stiffness matrix will contain 60 + 8 × 6 = 108 DOFs, with six additional DOFs per integration point to account for the characterization of inelastic strain. For a structure comprising *N* nodes and *M* elements, the total number of DOFs in the extended global stiffness matrix becomes N × 3 + M × 8 × 6. Although the stiffness matrix contains numerous zero entries and the use of sparse matrix solvers can alleviate efficiency losses to some extent, the substantial increase in DOFs still significantly hampers the solution process, particularly for large-scale models.

To address this inefficiency, we draw upon key insights from failure tests and numerical analyses of RC beams. The ultimate failure of a RC structure is typically governed by excessive crack widths in specific critical regions, such as the mid-span, pure bending zones, or shear-sensitive areas of beams, while the majority of concrete elements remain either uncracked or only minimally damaged during the loading process. Therefore, in a comprehensive structural analysis, it is unnecessary to apply the extended stiffness matrix formulation to all concrete elements. Instead, computational efficiency can be significantly enhanced by limiting the extended stiffness matrix modeling exclusively to cracked integration points within cracked elements. This approach allows the total number of DOFs in the global stiffness matrix to be dynamically adjusted according to the number of cracked integration points, rather than being fixed at the maximum possible value from the outset. The specific process for adaptive DOF setting is detailed as follows:

(1) Initialization: For a structure with *N* nodes and *M* elements (utilizing spatial 20-node solid elements), the initial number of DOFs in the global stiffness matrix is 3*N* (accounting for three displacement components per node), while each element’s initial stiffness matrix comprises 60 DOFs (consistent with conventional 20-node solid elements).
(2) After the first computation step: Suppose the structure develops one cracked integration point, denoted as *i*, located in element $m_{1}$. The total number of DOFs in the global stiffness matrix increases to 3*N* + 6. Consequently, the extended stiffness matrix of element $m_{1}$ now contains (60 + 6 = 66) DOFs, while all other elements retain their initial 60 DOFs.
(3) After *n* computation steps: If the structure accumulates *n* cracked integration points distributed across a set of elements $\left\{m_{1},m_{2},\cdots ,m_{I}\}(I\;\leq \;n\right)$, where multiple cracked integration points may reside within the same element, the total number of DOFs in the global stiffness matrix becomes 3N + 6n (with 6 additional DOFs per cracked integration point). For each element in the set $\left\{m_{1},{\;m}_{2},\;\cdots ,{\;m}_{I}\right\}$, the DOFs in its extended stiffness matrix are adjusted to 60 + 6m (where *m* is the number of cracked integration points within that element), while all other uncracked elements maintaining their original 60 DOFs.

This adaptive DOF strategy ensures that computational resources are concentrated solely on regions experiencing cracking, thereby minimizing unnecessary DOF expansion and significantly enhancing the efficiency of full-process cracking simulations for RC structures.

## 5. Numerical Examples

**Figure 2:**
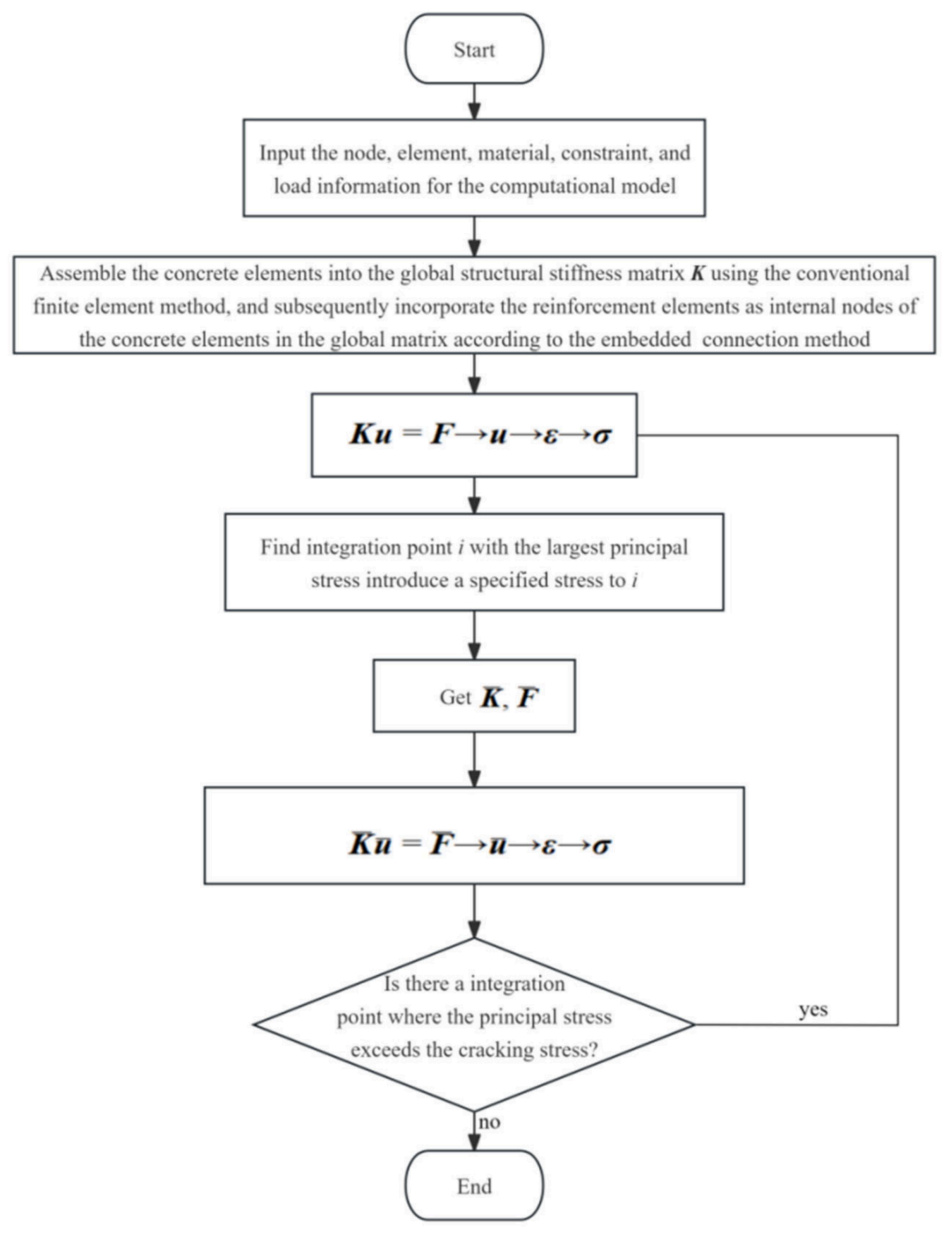
The flowchart.

To verify and examine the correctness and applicability of the proposed cracking algorithm, a corresponding finite element program was developed in C++ on the Visual Studio 2022 platform, and three numerical examples were designed for analysis. The detailed computational flowchart is shown in Figure 2. The first two examples are compared against theoretical solutions and experimental results, respectively, serving to validate the fundamental effectiveness of the proposed algorithm. The third example focuses on the crack development process in an under- reinforced beam, in which the crack distributions obtained by the proposed algorithm and by XFEM in ABAQUS 2022 are compared under identical modeling conditions, with the aim of illustrating the differences between the two methods in terms of their crack characterization capabilities. Through these numerical examples, particular attention is given to evaluating the performance of the proposed algorithm in simulating crack initiation, crack propagation paths, and the evolution of multiple cracks.

It is important to note that, in the simulation of cracking in RC members for this study, concrete is modeled using an elastic constitutive model, and environmental factors such as temperature are not taken into account. Therefore, in the subsequent numerical examples, the equivalent load corresponding to the known inelastic strain $\varepsilon^{p}$ [the second term on the right-hand side of Equation (13)] is set to zero within the calculation program.

### 5.1. RC Bar Under Uniform Tensile Load

**Figure 3:**
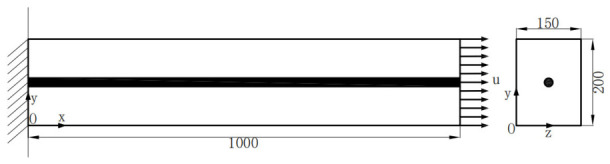
RC bar under uniform tensile load (unit: mm).

Example 1 focuses on verifying the fundamental correctness of the proposed algorithm through a simple yet representative model: a RC bar subjected to uniform tensile load, as shown in Figure 3. The material parameters for concrete and steel reinforcement are determined according to Chinese Standard [33]. The concrete material parameters are defined as follows: elastic modulus $E_{c}\;=\;30\;\mathrm{GPa}$, Poisson’s ratio $\nu_{c}\;=\;0.2$, and tensile strength $f_{t0}\;=\;2.01\;\mathrm{MPa}$. The steel reinforcement follows an ideal elastoplastic constitutive model with elastic modulus $E_{s}\;=\;200\;\mathrm{GPa}$, Poisson’s ratio $\nu_{s}\;=\;0.3$, yield strength $f_{y}\;=\;335\;\mathrm{MPa}$, and nominal diameter d = 12 mm.

Initially, the uncracked state was simulated to verify the consistency of the finite element results. An ABAQUS model was constructed using 8-node fully integrated solid elements (C3D8); due to the uniform stress field, only a single element was utilized. The boundary conditions were applied as follows: the four corner nodes at the left end were restrained in the *x*-direction, one lower corner node at the left end was additionally constrained in the *y*- and *z*-directions, and the other lower corner node was restrained in the *y*-direction. A uniformly distributed load $q\;=\;1\;\times \;10^{6}\;N/m^{2}$ was applied to the right end of the bar.

The displacements of the four corner nodes at the right end were calculated using both ABAQUS and the proposed algorithm, with the results summarized in Table 1 (where nodes 1 and 3 correspond to the upper two nodes at the right end, and nodes 2 and 4 correspond to the lower two nodes). U1, U2, and U3 represent the displacements in the x, y, and z directions, respectively. As shown in Table 1, the results from the proposed algorithm are in excellent agreement with those obtained from ABAQUS, thereby confirming the reliability of the algorithm in simulating elastic behavior.

Since the stress is uniform at all points in the tension bar illustrated in Figure 3, if concrete is assumed to be a homogeneous material, the member would crack simultaneously at every point upon reaching the cracking load. To simulate the heterogeneity of concrete and more effectively illustrate the crack propagation path modeled by the proposed algorithm, a function f(x,y,z) was introduced to characterize the spatial variability of concrete tensile strength. For simplicity, the function is defined as

$$f\left(x,y,z\right)=\frac{x+y+z}{3X}\;X=\max({\left|x_{i}\right|}_{\max},{\;\left|y_{i}\right|}_{\max},\;{\left|z_{i}\right|}_{\max})$$

where $x_{i},{\;y}_{i},\;z_{i}$ are the coordinates of point i within the structure. The tensile strength at any point in the structure can then be expressed as

(22) $$f_{t}\left(x,y,z\right)=\;f_{t0}(1+f\left(x,y,z\right))$$

The tensile strengths and corresponding cracking displacement loads of the eight integration points are presented in ascending order in Table 2. The cracking displacement loads in Table 2 are calculated using the geometric equation $\varepsilon_{c}\;=\;u/L$ and the physical equation $f_{t}\;=\;E_{c}\varepsilon_{c}$. Four loading cases were selected based on these cracking displacement loads to simulate the cracking process of the tension bar, with the number of cracked integration points and crack propagation paths summarized in Table 3.

Key observations can be drawn from Table 2 and Table 3: when the applied displacement load of 5.70 × 10^−5^ m is lower than the cracking displacement load of all integration points, no cracking occurs. Conversely, when the load of 9.5 × 10^−5^ m exceeds the cracking displacement load of all integration points, full-element cracking is observed. Additionally, the crack propagation path and the sequence of cracked integration points closely correspond to the ranking of tensile strengths and cracking displacement loads presented in Table 2. This finding suggests that the proposed algorithm can simulates the cracking behaviors in heterogeneous RC tension members.

**Figure 4:**
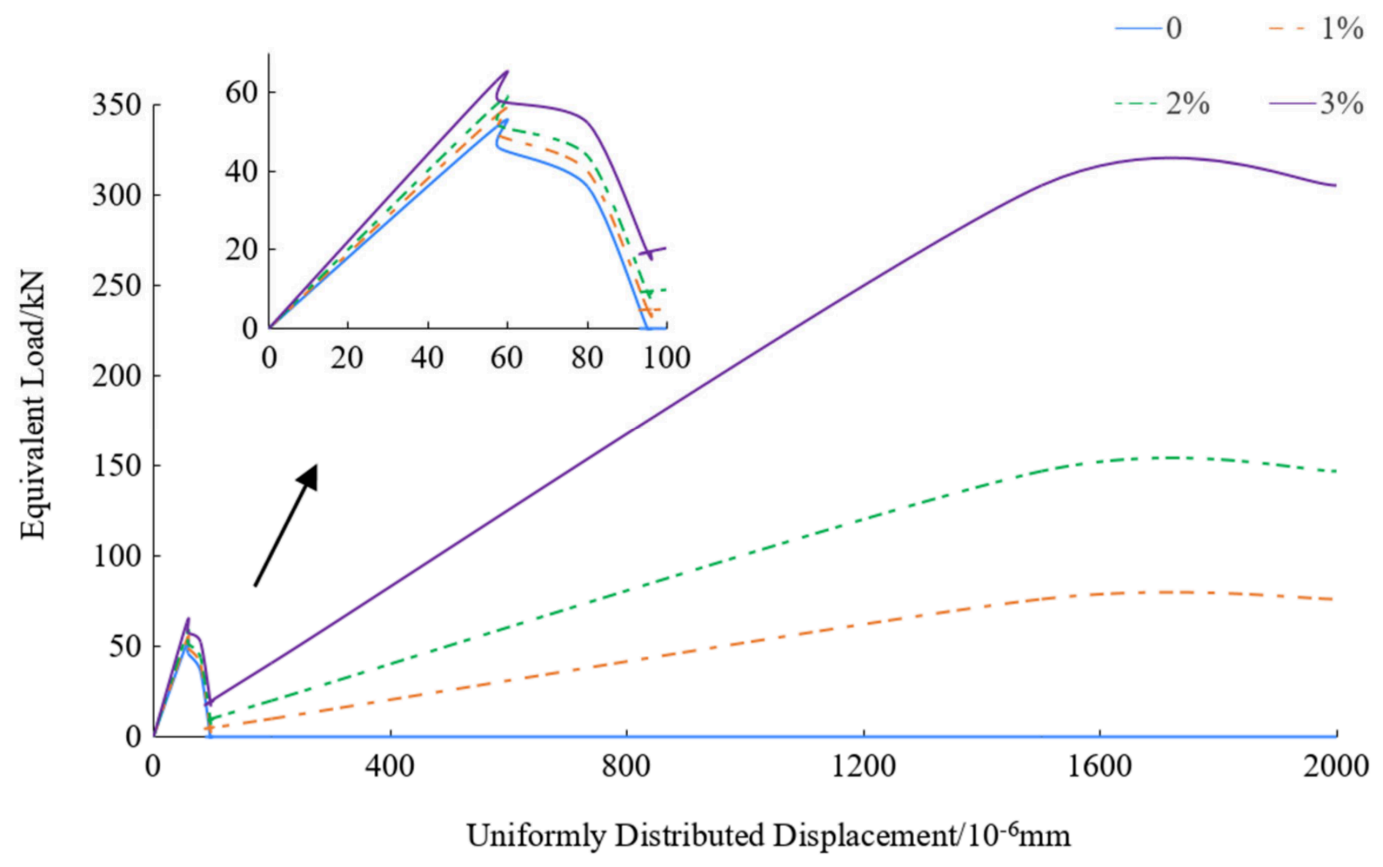
Load–displacement curves at the right end of the RC bar.

Figure 4 illustrates the load–displacement curves at the right end of the RC bar for various reinforcement ratios (ρ = 0%, 1%, 2%, 3%). The enlarged view presents the load–displacement curve corresponding to the moment at which all integration points of the RC bar just reach the fully cracked state. The curves demonstrate consistent developmental trends across all reinforcement ratios: due to the elastic constitutive model of concrete, the load–displacement relationship remains linear until the cracking load is reached (elastic stage). Following cracking, the load-carrying capacity experiences a rapid decline. As the number of cracked integration points increases, the rate of capacity reduction diminishes (stable crack propagation). Once all integration points have cracked, the load-carrying capacity of the unreinforced bar drops to zero, whereas the load in the reinforced bar is primarily carried by the reinforcement. As displacement increases, the load rises approximately linearly until the reinforcement yields. This behavior aligns with theoretical expectations of crack propagation in RC bars, further validating the algorithm.

Moreover, as the reinforcement ratio increases, the structural stiffness, cracking load, and residual load-carrying capacity (after full cracking) all exhibit an upward trend—reflecting the reinforcing effect of steel bars and confirming the algorithm’s capability to capture the influence of reinforcement on cracking behavior.

### 5.2. RC Beam Under Mixed-Mode Cracking

**Figure 5:**
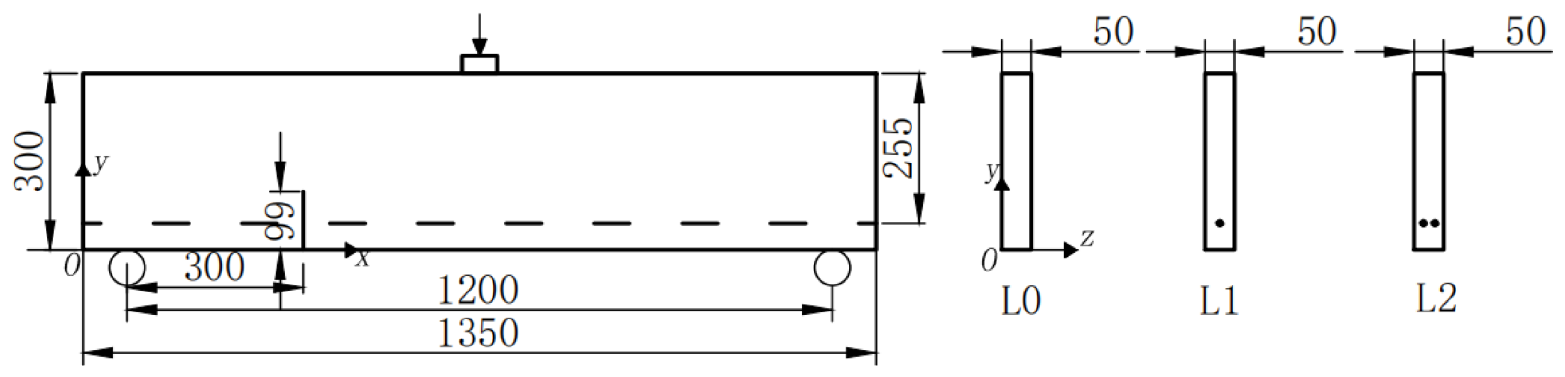
Dimensions and reinforcement details of the pre-formed beam.

Example 2 aims to verify the algorithm’s accuracy and applicability in simulating mixed-mode (tension-shear) cracking in RC beams, referencing the experimental setup by Carmona et al. [34]. The beam dimensions are as follows: length = 1350 mm, width = 50 mm, height = 300 mm, span = 1200 mm, and a pre-formed crack measuring 99 mm in length, located 300 mm from the left support, as shown in Figure 5. The steel reinforcement has a nominal diameter of d = 2.5 mm and a yield strength of $f_{y}$ = 563 MPa, with elastic modulus $E_{s}\;=\;174\;\mathrm{GPa}$, Poisson’s ratio $\nu_{s}\;=\;0.3$; the concrete parameters include elastic modulus $E_{c}\;=\;28.3\;\mathrm{GPa}$, Poisson’s ratio $\nu_{c}\;=\;0.2$, compressive strength $f_{c}$ = 36.3 MPa, tensile strength $f_{t}$ = 3.8 MPa and fracture energy $G_{f}$ = 43.4 N/m. Three beam types are considered: L0 (plain concrete beam), L1 (RC beam with one longitudinal bar), and L2 (RC beam with two longitudinal bars).

An ABAQUS model was developed using 20-node reduced integration solid elements (C3D20R) for comparison purposes. The boundary conditions are as follows: the left support is fixed in the *x*-, *y*-, and *z*-directions, while the right support is restrained in the *y*- and *z*-directions. To prevent stress concentration and enhance numerical convergence, a uniformly distributed displacement load was applied over a small area (20 mm × 50 mm) at the mid-span, which is equivalent to a concentrated load.

**Figure 6:**
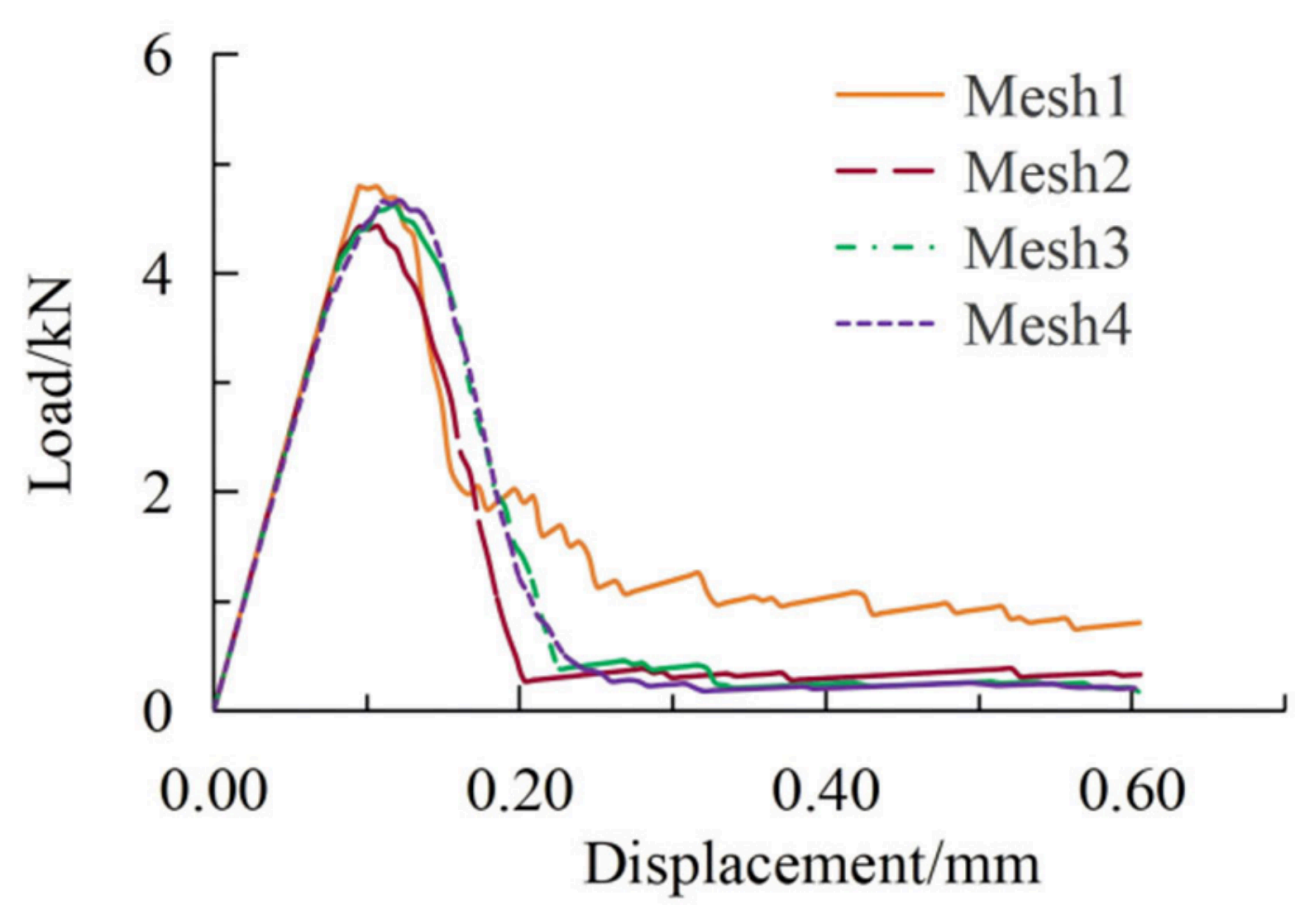
Load–displacement curves of plain concrete beam L0 under different meshing schemes.

First, four meshing schemes, denoted as Mesh1, Mesh2, Mesh3, and Mesh4, were employed to evaluate the mesh sensitivity of the plain concrete beam L0. Table 4 summarizes the ultimate loads obtained using the four meshes, and Figure 6 presents the corresponding load–displacement curves. Mesh1 represents a relatively coarse discretization and shows a noticeable difference from the finer meshes. As the mesh is refined, the relative difference in the ultimate load decreases from 8.1% between Mesh1 and Mesh2 to 4.1% between Mesh2 and Mesh3, and further to 0.94% between Mesh3 and Mesh4. Moreover, the load–displacement curves obtained using Mesh3 and Mesh4 are close in terms of the peak load, peak displacement, and overall post-peak response. This indicates that the numerical results tend to stabilize when the element size is reduced to 20 mm or smaller. Therefore, Mesh3 was adopted in the subsequent analyses by considering both computational accuracy and efficiency.

**Figure 7:**
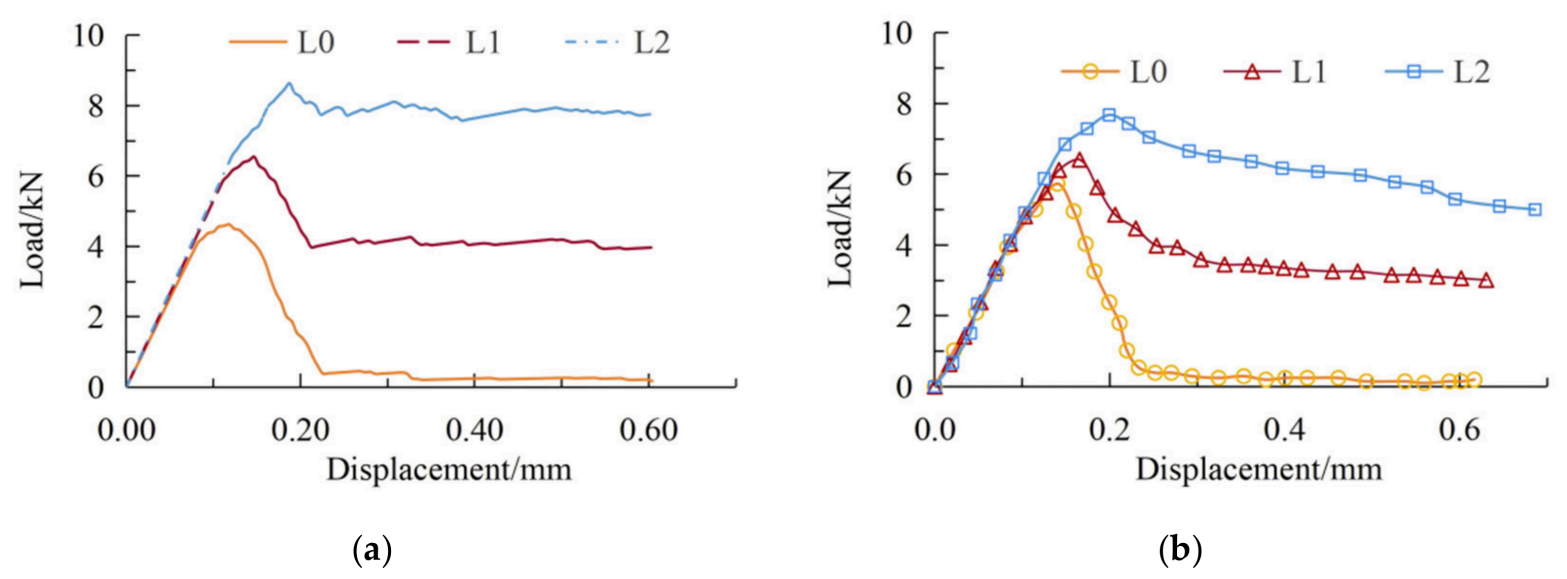
Comparison of load–displacement curves for Example 2: (**a**) numerical results obtained using the proposed cracking algorithm; (**b**) experimental curves redrawn by the authors based on digitized data extracted from Ref. [34].

Figure 7a presents the load–displacement curves at the loading point for the plain concrete beam (L0), the RC beam with one longitudinal rebar (L1), and the RC beam with two longitudinal rebars (L2), obtained using the cracking algorithm proposed in this paper. Figure 7b shows the corresponding experimental load–displacement curves for these specimens. The experimental data were digitized from the load–displacement curves reported in Ref. [34] and redrawn by the authors for comparison.

A comparison between Figure 7a,b indicates that the load–displacement responses predicted by the proposed algorithm follow trends that are generally consistent with the experimental results. This suggests that the algorithm is able to capture the main trends of the load–displacement behavior of RC beams under composite cracking modes.

It should be noted that the cracking criterion adopted in this paper is based on the maximum principal stress at the integration point reaching the tensile strength and does not account for fracture-energy effects. Therefore, the calculated ultimate load for the plain concrete beam (L0) is lower than the experimental value.

**Figure 8:**
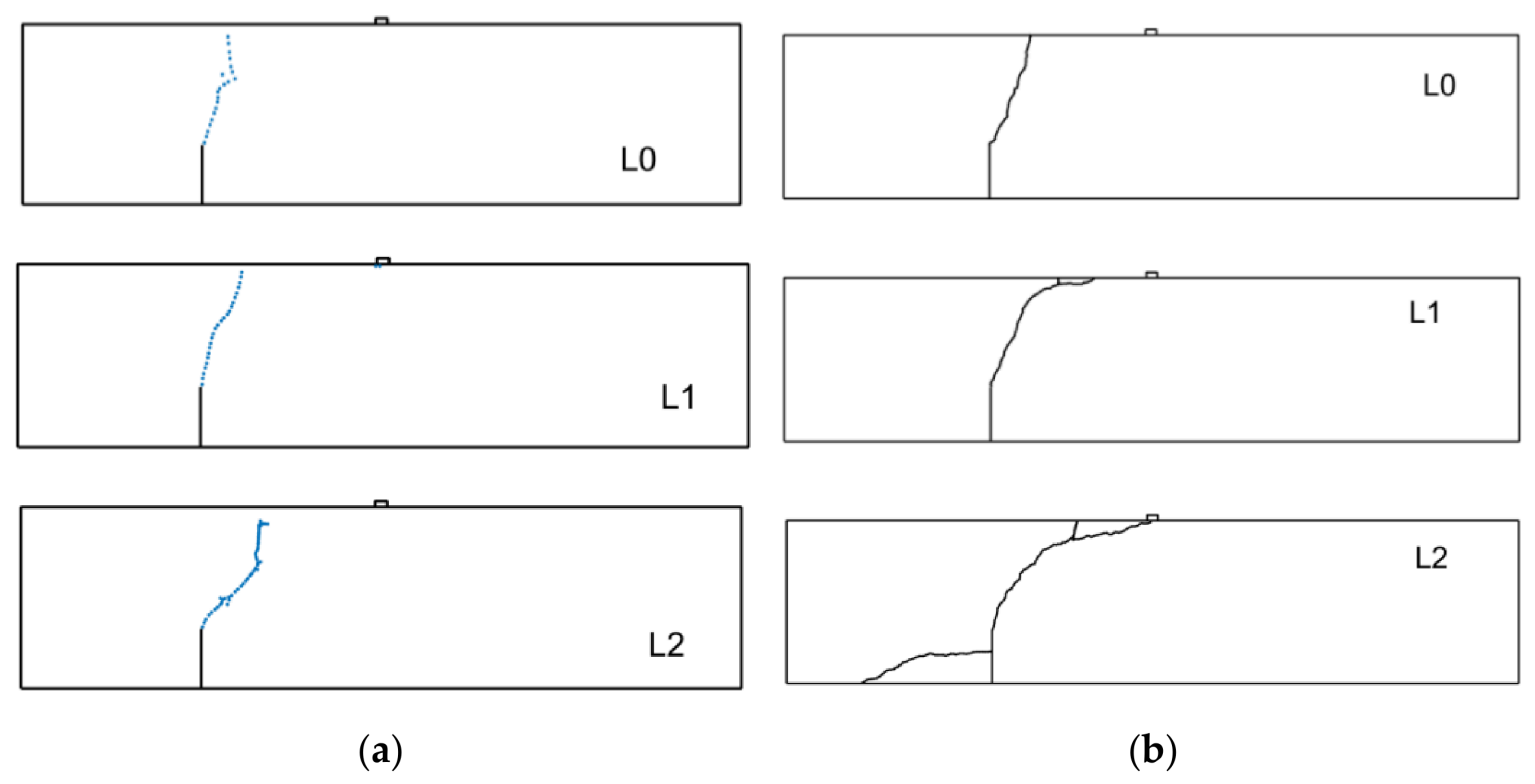
Comparison of crack propagation patterns: (**a**) results from the present study; (**b**) experimental crack patterns of specimens L0, L1, and L2 digitized from Ref. [34] and redrawn by the authors.

In this study, a numerical program written in C++ is used to compute key data, including the coordinates of cracked integration points, which are subsequently visualized in MATLAB R2018b to determine the crack propagation path. Figure 8a illustrates the crack propagation state, based on the cracked integration points calculated using the cracking algorithm proposed in this study. Figure 8b presents the experimentally observed crack patterns of specimens L0, L1, and L2, which were digitized from Ref. [34] and redrawn by the authors. Specifically, L0 denotes the plain concrete beam, while L1 and L2 denote the RC beams with one and two longitudinal bars, respectively.

As shown in Figure 8, under combined tension-shear action, cracks in the plain concrete beams (L0) initiate from the tip of the pre-existing notch and propagate almost linearly toward the loading point at a small inclination angle, ultimately leading to beam failure. Due to the relatively weak shear resistance of plain concrete, the cracking process is predominantly governed by Mode I (opening) cracking, resulting in a relatively straight crack path.

For the RC beams, the steel bars begin to participate in load-bearing from the initial loading stage. As the reinforcement ratio increases, the shear force carried by the longitudinal bars also increases, thereby enhancing the dominance of Mode II (sliding) cracking during the crack initiation phase. Consequently, with an increasing reinforcement ratio, the crack propagation path exhibits a more pronounced curved characteristic, and the intersection point of the crack with the top surface of the beam gradually moves closer to the loading point.

A comparison between Figure 8a,b reveals that the crack propagation patterns simulated by the proposed algorithm are generally consistent with the experimental results, indicating that the algorithm can simulate the mixed-mode cracking process in RC beams. Nevertheless, in line with the above observation on the load–displacement response, this agreement, particularly for the crack patterns, should presently be regarded as qualitative, since fracture energy effects and bond–slip behavior are not accounted for in the current formulation. Moreover, it should be noted that the cracking criterion adopted in the algorithm presented in this paper is relatively simple, which leads to a certain dependence of the crack path on the mesh configuration. Future studies may incorporate more realistic cracking criteria to improve the algorithm and further enhance its computational robustness and reliability.

During the experimental loading process, the increase in tensile stress within the longitudinal reinforcement raises the bond stress between the steel and concrete. Furthermore, the “dowel action” of the longitudinal reinforcements may induce bond cracks or splitting cracks along the reinforcement, as observed in the multiple cracking phenomena in beam L2 shown in Figure 8b. The simulation in this study adopts a perfect steel-concrete bond assumption and neglects bond-slip and splitting effects, hence failing to reproduce these phenomena.

### 5.3. Multiple-Crack Propagation in an Under-Reinforced RC Beam

**Figure 9:**
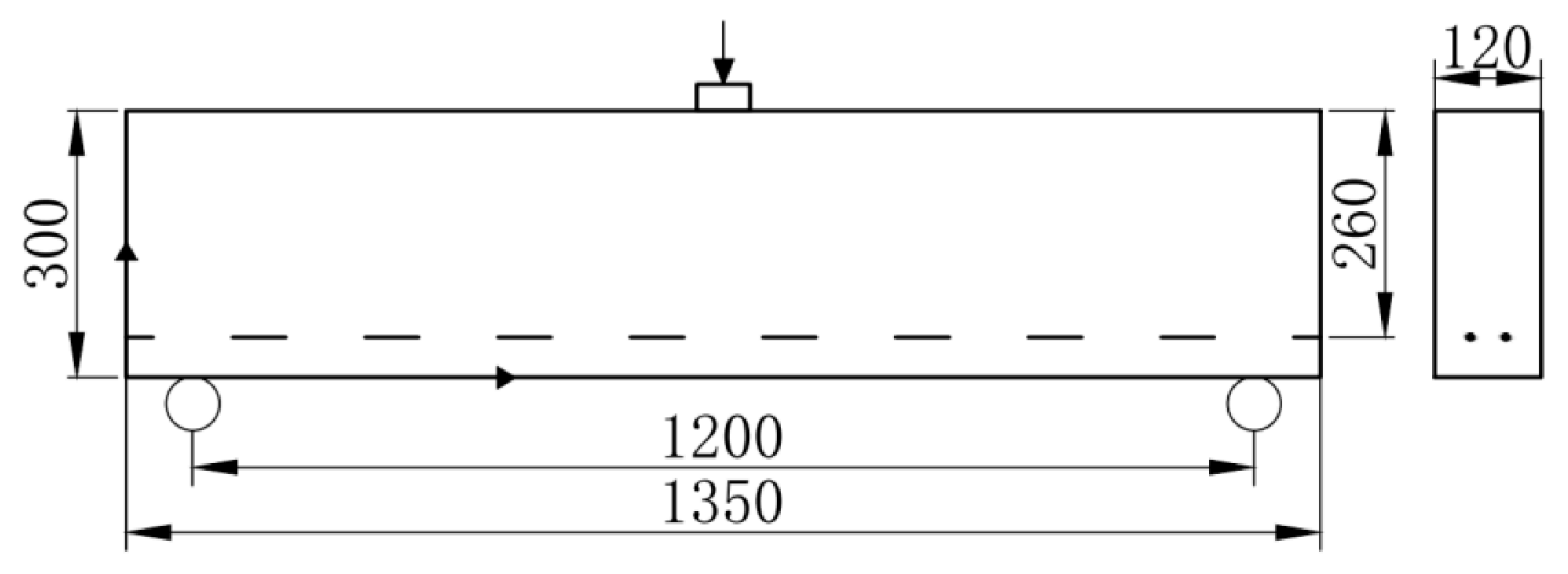
Schematic diagram of the RC simply supported beam.

Example 3 presents a comparative study of multi-crack propagation in an under-reinforced RC beam, with the aim of illustrating the crack characterization capabilities of the proposed algorithm relative to the XFEM module in ABAQUS. A cracking simulation is conducted on an under-reinforced RC beam (reinforcement ratio of 1.28%), subjected to a concentrated load at mid-span, as illustrated in Figure 9. The material parameters for concrete and steel reinforcement are taken from reference [35] as follows: the steel reinforcement has a nominal diameter of d = 14 mm and a yield strength of $f_{y}$ = 583 MPa, with elastic modulus $E_{s}\;=\;210\;\mathrm{GPa}$, Poisson’s ratio $\nu_{s}\;=\;0.3$; the concrete parameters include elastic modulus $E_{c}\;=\;31.5\;\mathrm{GP}$, Poisson’s ratio $\nu_{c}\;=\;0.2$, compressive strength $f_{c}$ = 22.54 MPa, tensile strength $f_{t}$ = 2.14 MPa and fracture energy $G_{f}$ = 120 N/m.

A finite element model of the RC beam was established in ABAQUS, and the XFEM algorithm—which in ABAQUS is restricted to first-order elements—was employed using C3D8R elements to simulate crack propagation under different load levels. The results were compared with those obtained from the proposed algorithm. To demonstrate the applicability of the proposed algorithm to higher-order elements, a 20-node reduced integration element (C3D20R) was adopted in the program to simulate crack propagation in the under-reinforced RC beam. Furthermore, to mitigate stress concentration and improve convergence during the cracking calculation, a uniformly distributed displacement load was applied over a small region (20 mm × 50 mm) at mid-span as an equivalent representation of the concentrated mid-span load.

**Figure 10:**
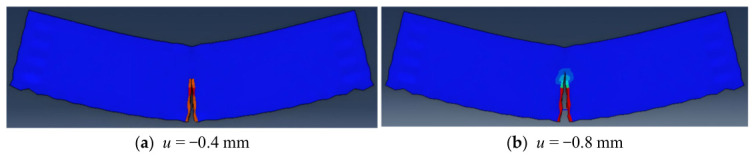
Crack propagation pattern of the simply supported beam simulated using XFEM.

**Figure 11:**
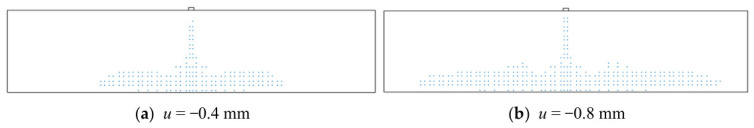
Crack propagation pattern of the simply supported beam simulated using the specified stress method.

Figure 10a,b depict the crack propagation patterns of the RC simply supported beam calculated using the XFEM algorithm in ABAQUS under uniformly distributed displacement loads at mid-span of u=−0.4 mm and u=−0.8 mm, respectively. These two displacement levels are considered to illustrate the progressive development of cracks in the under-reinforced RC beam as the applied displacement increases, including the increase in crack number and crack height. In this regard, the response at u=−0.8 mm should be regarded as a continuation of that at u=−0.4 mm. Figure 11a,b present the corresponding crack propagation patterns based on the cracked integration points obtained from the proposed algorithm under the same displacement loads, using a displacement increment of 0.008 mm.

As observed in Figure 10, under the concentrated load at mid-span, a single crack initiates at the bottom center of the simply supported beam and propagates upward as the load increases, with no new cracks developing on either side of the mid-span. In contrast, Figure 11 reveals that the crack pattern simulated by the proposed algorithm under the same loading condition is characterized by a taller primary crack at the bottom center of the mid-span, accompanied by several short, fine cracks that appear almost symmetrically on both sides of mid-span. With increasing load, the height of the primary crack increases, and more fine cracks emerge on both sides of the mid-span, all exhibiting varying degrees of height growth.

It should be noted that the XFEM result obtained from ABAQUS is used only as a comparative reference to illustrate the difference in the predicted crack propagation patterns. Due to the limitation of the element formulation adopted in the XFEM analysis, the XFEM result is not intended to serve as a strict quantitative benchmark for evaluating the accuracy of the proposed method. Comparing Figure 10 and Figure 11, it can be observed that the XFEM simulation in ABAQUS mainly captures one dominant crack path near the mid-span of the beam. In contrast, the proposed method predicts several cracking/damage zones initiating from the bottom tensile region and propagating upward, together with a dominant flexural crack near the loading region. This behavior appears qualitatively closer to the expected flexural cracking behavior of under-reinforced RC beams, in which multiple cracks generally develop in the tensile zone before failure.

In the proposed method, the crack propagation pattern is identified from the spatial distribution of cracked integration points satisfying the cracking criterion. Therefore, the plotted pattern reflects the evolution of crack paths within the finite element discretization. Although the cracks are not represented as explicitly inserted geometrical discontinuity surfaces, the proposed method can capture the initiation, propagation, and spatial distribution of multiple cracking/damage zones in the tensile region of the under-reinforced RC beam.

Furthermore, unlike the XFEM algorithm in ABAQUS, which is limited to first-order elements, the proposed algorithm can utilize second-order elements (e.g., C3D20R) for crack propagation calculations. Second-order elements may provide improved stress accuracy over first-order elements; under otherwise identical computational conditions, this could potentially allow for a more refined identification of cracking regions and patterns, thus enabling a more detailed cracking analysis. However, it should be emphasized that the improved stress accuracy does not necessarily translate into quantitatively superior crack prediction without experimental validation.

**Figure 12:**
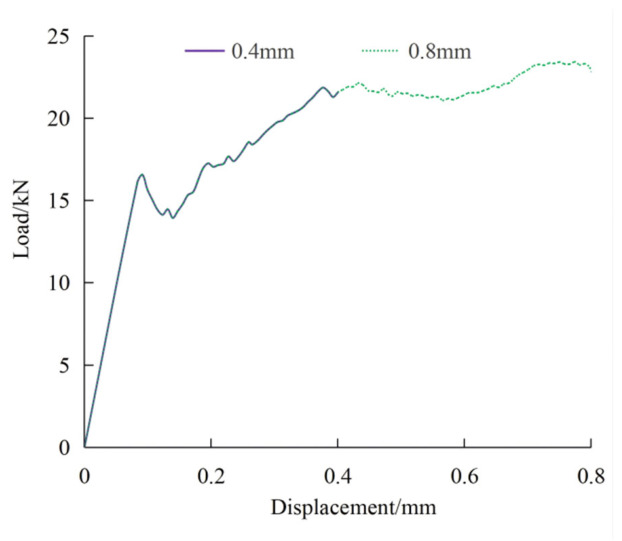
Load–displacement curves of an under-reinforced RC beam obtained using the proposed algorithm.

Figure 12 presents the load–displacement curves at the loading point of an under-reinforced RC beam obtained using the proposed algorithm under two mid-span displacement loading conditions (u=−0.4 mm and u=−0.8 mm). The figure shows that the load–displacement curves from the proposed method can capture the typical mechanical behavior of under-reinforced RC beams. Prior to concrete cracking, the load–displacement relationship is linear with a relatively steep slope. After cracking, the bearing capacity exhibits brief fluctuations and the structural stiffness decreases. As the cracking process progresses, the slope of the load–displacement curve gradually diminishes while the load continues to increase. Eventually, after the reinforcement yields, the curve flattens out and develops in an almost horizontal manner. These results suggest that the proposed algorithm is capable of simulating the multiple crack propagation process in under-reinforced RC beams and appears to provide a reasonable representation of their load–displacement response.

It should be noted that, although the specified-stress method employed in this paper is capable of capturing the qualitative distribution characteristics of multiple cracks, its application to crack simulation is still in an exploratory stage. The predicted crack patterns reflect the spatial distribution of cracked integration points rather than explicitly resolved crack geometries, and the method cannot yet provide quantitatively accurate reproduction of the detailed crack morphology observed experimentally. Further research will be carried out, building on references [36,37], to refine the crack simulation.

### 5.4. Effectiveness of the Adaptive DOF Strategy

To quantify the computational benefit of the adaptive degree-of-freedom strategy described in Section 4, the numbers of degrees of freedom (DOFs) involved in Examples 1 and 3 at different cracking stages are summarized in Table 5. For each example, the number of additional inelastic-strain DOFs activated by the adaptive strategy is compared with that required by a hypothetical full-field allocation, in which the additional unknowns are assigned to all integration points of the model.

In the proposed framework, six additional inelastic-strain unknowns are introduced for each cracked integration point, and these unknowns are activated only where the crack-plane stress condition is enforced. Consequently, the number of additional DOFs grows strictly linearly with the number of cracked integration points, as illustrated by Example 1, in which the additional DOFs increase from 6 at crack initiation to 48 when all integration points have cracked. It should be emphasized that the adaptive strategy is an exactness-preserving implementation technique rather than an approximation: since the additional unknowns are simply not needed at uncracked integration points, the computed results are identical to those that would be obtained with full-field allocation, and only the size of the resulting system of equations differs.

The practical benefit of the strategy becomes evident in large-scale models. In Example 3, a full-field allocation would require 16,848 additional DOFs, more than twice the number of standard displacement DOFs (8100). With the adaptive strategy, only 6 additional DOFs are activated at crack initiation; as cracking develops within each load case, the number of activated DOFs reaches 2460 for the displacement level of u=−0.4 mm and 5376 for the larger displacement level of *u* = −0.8 mm, corresponding to 14.6% and 31.9% of the full allocation, respectively. In other words, even at the larger displacement level considered (*u* = −0.8 mm), the adaptive strategy thus reduces the number of additional unknowns by approximately 68%, and the total problem size (13,476 DOFs) is only about 54% of that required by full-field allocation (24,948 DOFs). These results confirm that the additional computational cost of the proposed method scales with the extent of cracking rather than with the total model size.

A systematic assessment of the computational performance of the framework, including runtime and memory usage based on an optimized implementation, will be conducted in future work, as noted in Section 6.

## 6. Conclusions

Based on the Specified Stress Method, this study proposes a finite element computational scheme for RC member crack analysis and develops a corresponding calculation program in C++. The feasibility and potential applicability of the proposed algorithm were examined through three numerical examples involving RC members under different loading conditions and structural forms.

The results indicate that the algorithm is able to simulate crack initiation and propagation in RC members while accounting for the heterogeneity of concrete materials, and that it can reasonably capture the mixed-mode cracking process of pre-formed RC beams under tension–shear coupling. Furthermore, unlike the XFEM module in ABAQUS—which is restricted to first-order elements and produced only a single dominant crack in the comparative example examined herein—the proposed algorithm allows for the use of second-order elements and is capable of capturing multiple distributed cracking zones in under-reinforced RC beams, a pattern qualitatively consistent with well-established flexural cracking behavior.

In the proposed method, inelastic strain is introduced as an additional unknown, and concrete cracking is represented by specifying the stress on the crack surface to be zero. After cracking, the crack-surface stress is maintained at zero, thereby avoiding the issue of damage reversibility. The method exhibits reasonable convergence during the computational process and requires neither a predefined crack path nor remeshing after crack formation. Based on the computed crack distribution and propagation process, the crack geometry and its evolution characteristics can be reasonably described. The numerical results indicate that the cracking simulation framework based on the Specified Stress Method can reasonably capture the principal cracking features and overall mechanical response of the RC members investigated in this study. This work indicates the feasibility of applying the Specified Stress Method to the simulation of cracking in RC members and provides a complementary computational framework for existing numerical approaches to crack analysis of RC structures.

At the same time, the present study still has scope for further extension and refinement. Future research may be conducted in the following aspects:

(1) Extension of concrete constitutive models: The concrete material is currently described using a linear elastic constitutive model, which is sufficient for verifying the basic computational mechanism of the specified stress method in crack simulation. To further enhance the capability of the proposed method in capturing the nonlinear mechanical behavior of RC members, elastoplastic or plastic-damage constitutive models may be incorporated into the specified stress framework in future work. This would enable a more refined representation of stress evolution, stiffness degradation, and post-peak response of concrete before and after cracking.
(2) Optimization of cracking criteria and simulation methods: The current algorithm adopts the maximum tensile stress criterion and represents crack formation by setting the stress at cracked integration points to zero. This treatment is capable of describing the crack initiation and propagation processes in the numerical examples considered in this study; however, the predicted crack path may still be affected by the mesh configuration. In future work, fracture energy-based regularization and fracture mechanics-based criteria may be incorporated to mitigate potential mesh sensitivity and to predict the crack propagation direction. In addition, non-regularized methods [13,14] may be introduced to improve the algorithm, and measures such as the definition of cracked elements may be adopted to further enhance the stability and accuracy of crack path simulation.
(3) Consideration of bond-slip between reinforcement and concrete: In the current algorithm, a deformation compatibility assumption is adopted between the reinforcing bars and the surrounding concrete, and the bond-slip behavior between them has not yet been considered. For problems involving stringent requirements for the prediction of crack width, stress redistribution in reinforcement, and post-peak response, the bond-slip effect may have a significant influence on the computational results. In future work, approaches such as the lattice discrete particle model [37] and non-nodal coupling techniques [38] may be introduced to describe the interaction between reinforcement and concrete, thereby further improving the simulation accuracy of both local cracking behavior and the overall mechanical response of RC members.

Systematic quantitative validation against additional experimental datasets and established numerical approaches will be conducted in future work, after more advanced constitutive models and bond–slip models are incorporated into the framework. A systematic assessment of the computational efficiency of the proposed framework—including runtime, memory usage, and convergence behavior in comparison with established methods such as XFEM and phase-field models—will be conducted in future work, based on a further optimized implementation.

## Figures and Tables

**Table 1:** Comparison of calculation results of finite element displacement (unit: m).

Node	U1		U2		U3	
Number	ABAQUS	The Present	ABAQUS	The Present	ABAQUS	The Present
1	3.25 × 10^−5^	3.25 × 10^−5^	−1.30 × 10^−6^	−1.30 × 10^−6^	−3.45 × 10^−14^	−2.44 × 10^−20^
2	3.25 × 10^−5^	3.25 × 10^−5^	−1.69 × 10^−14^	−4.52 × 10^−20^	−3.44 × 10^−14^	−2.40 × 10^−20^
3	3.25 × 10^−5^	3.25 × 10^−5^	−1.30 × 10^−6^	−1.30 × 10^−6^	9.75 × 10^−7^	9.75 × 10^−7^
4	3.25 × 10^−5^	3.25 × 10^−5^	−1.69 × 10^−14^	−4.63 × 10^−20^	9.75 × 10^−7^	9.75 × 10^−7^

**Table 2:** Tensile strengths and cracking displacement loads at integration points.

Integration Point Number	Strength/Pa	Cracking Displacement Load/m
7	1.72 × 10^6^	5.74 × 10^−5^
5	1.84 × 10^6^	6.13 × 10^−5^
3	1.88 × 10^6^	6.26 × 10^−5^
1	1.99 × 10^6^	6.64 × 10^−5^
8	2.50 × 10^6^	8.32 × 10^−5^
6	2.61 × 10^6^	8.71 × 10^−5^
4	2.65 × 10^6^	8.84 × 10^−5^
2	2.77 × 10^6^	9.22 × 10^−5^

**Table 3:** Number of cracked integration points and crack propagation paths under four loading cases.

	Displacement Load/m			
	5.70 × 10^−5^	5.80 × 10^−5^	8.00 × 10^−5^	9.5 × 10^−5^
Number of Crack Integral Points	0	1	4	8
Crack Propagation Path	-	7	7→5→3→1	7→5→3→1→8→6→4→2

**Table 4:** Ultimate load of plain concrete beam L0 under different mesh configurations.

Grid Scheme	Element Size/mm	Number of Elements	Ultimate Load/kN	Relative Error/%
Mesh1	40	324	4.79	
Mesh2	30	546	4.43	8.1
Mesh3	20	1176	4.62	4.1
Mesh4	15	2150	4.66	0.94

**Table 5:** Effectiveness of the adaptive degree-of-freedom strategy (Examples 1 and 3).

Indicator	Example 1 (Initial→Final Cracking)	Example 3 (Initial→Final Cracking)	
		*u* = −0.4 mm	*u* = −0.8 mm
Standard Displacement DOFs	24	8100	8100
Additional DOFs, Full Allocation	48	16,848	16,848
Additional DOFs Activated (Adaptive)	6→48	6→2460	6→5376
Activated/Full Allocation	12.5%→100%	0.036%→14.6%	0.036%→31.9%

## Data Availability

The original contributions presented in this study are included in the article. Further inquiries can be directed to the corresponding author.
